# Mirabegron exhibits antibacterial and Immunomodulatory activities demonstrated by in vitro and in vivo studies

**DOI:** 10.1038/s41598-025-23392-2

**Published:** 2025-11-07

**Authors:** Esraa A. Ahmed, Ahmed M. Abd-Eldayem

**Affiliations:** 1 Pharmacology Department, Faculty of Medicine, Assiut University, Assiut, Egypt; 2 Department of Basic Sciences, College of Medicine, Fahad Bin Sultan University (FBSU), 15700, Tabuk, 71454 Kingdom of Saudi Arabia

**Keywords:** Mirabegron, Bacterial growth, Immune response, In vitro, In vivo, Biological models, Immunological techniques, Microbiology techniques, Medical research

## Abstract

Mirabegron is a beta 3-adrenoceptor agonist that can help with incontinence, dysuria, and bladder overactivity. It alleviates the symptoms of prostate enlargement and urinary tract infections (UTIs) in the elderly. We aimed to explore Mirabegron’s antimicrobial and immune-boosting properties, trying to benefit people suffering from urinary tract infections and having an overactive bladder. Mirabegron’s putative antibacterial activity was investigated using the well diffusion method (In Vitro). Infected rats were treated with mirabegron (10 mg/kg, oral) and used for evaluation of immunomodulatory actions (In Vivo). We tested the antibacterial activity in vitro against numerous bacterial strains, including *Escherichia coli* (*E. coli)*. The phagocytic activity and survival of peritoneal macrophages were examined. Also, serum levels of immunoglobulin G (IgG), immunoglobulin M (IgM), and interferon-γ (INF-γ) were estimated in *E. coli*-infected rats. We found that mirabegron exhibited significant antibacterial activity, particularly against *E. coli*. Phagocytic activity increased notably in vivo, indicating an improved innate immune response. Mirabegron also demonstrated a substantial rise in immunoglobulin and cytokine levels, enhancing acquired immunity. According to our findings, in vitro and in vivo tests of mirabegron revealed possible antibacterial activity as well as immunomodulatory properties. Mirabegron could alleviate symptomatic UTIs.

## Introduction

Mirabegron is a selective beta 3-adrenoceptor agonist^1^ and one of several novel medicines used for managing overactive bladder (OAB). Beta 3-adrenoceptor stimulation helps the bladder become full and hold urine, but it does not block bladder-voiding contractions^1,2^. Mirabegron reduces both the frequency of micturition and the number of daily incontinence episodes^3–5^. Mirabegron was associated with fewer adverse effects, and it is more effective when the degree of urine incontinence rises^6^. Increased heart rate and blood pressure are uncommon adverse effects; however, they tend to fade off in patients with OAB^6,7^. Mirabegron is an effective, safe, and beneficial drug for men suffering from OAB and benign prostatic hyperplasia (BPH)^8^. Mirabegron is suggested for adults at a daily dosage of 50 mg ^9,10^. It is metabolized by many routes, including cytochrome P450^11–13^.

Behavioral therapy is the primary line of treatment for OAB, followed by β-3 adrenergic agonists, antimuscarinics (AM), and combination therapy^14^. Mirabegron, a β3-adrenoceptor agonist, may improve durability and adherence to oral OAB pharmacotherapies and has been recommended by the American Urology Association/Society of Urodynamics, Female Pelvic Medicine, and Urological Reconstruction (AUA/SUFU) and authorized by the FDA as a combination therapy for OAB^15,16^. A prospective observational study was conducted, and mirabegron showed the biggest improvement in OAB-V8 scores in patients with OAB syndrome. The mirabegron group had fewer side effects than the solifenacin group^17^. Mirabegron is utilized as the first line of therapy for OAB due to its decreased adverse effect profile^18^.

Overactive bladder shares symptoms with other urological conditions like UTIs, BPH, and bladder cancer in situ, such as urgency, frequency, and nocturia^19^. While *E. coli* is the leading cause of UTIs^20,21^, the etiology of OAB is multifactorial^22,23^. Disruption of the urinary and gut microbiota contributes to bladder disease pathogenesis and may exacerbate OAB symptoms^24–26^. Low-grade bacterial presence in the bladder can worsen OAB and increase UTI frequency, especially in cases of compromised host immunity^27^. Evidence is mixed on whether anti-muscarinic agents and β3-adrenoceptor agonists increase UTI risk; some studies suggest they might, while others report no significant association^27–29^.

Distinct urinary microbiota patterns are observed in OAB patients with detrusor overactivity, suggesting that specific bacterial profiles may be linked to this condition. These findings underscore the potential of urinary microbiota analysis for more precise diagnosis and targeted treatment of OAB subtypes^30^. Urinary chronic pelvic pain syndrome (UCPPS) has been associated with alterations in both urinary and gut microbiota. Reduced diversity and imbalanced composition of the gut microbiota may play a role in its pathogenesis. Shifts in specific urinary microbial populations support a microbial contribution to UCPPS^31^. Newer technologies, such as RNA sequencing and extended culture methods, demonstrate that urine is not sterile, and organisms found in urine may be the source of OAB symptoms^32^. In treatment-resistant OAB, undetected UTIs might be a hidden cause. Urothelial inflammation and bacterial colonization heighten the sensitivity of bladder sensory neurons to distension, intensifying core OAB symptoms like urgency, frequency, and nocturia^33^. In OAB patient samples, *E. coli* exhibited a stronger association with urothelial cells in sediment cultures, which was not seen in controls^34^.

The nervous system plays a crucial role in regulating immune cell function, inflammation, and cytokine levels^35,36^. The immune system’s many cells function on a sliding scale, from the innate immune system to highly specialized responses in the adaptive immune system^37^. Sympathoadrenergic fibers supply both primary and secondary lymphoid organs, where noradrenaline and adrenaline target adrenergic receptors on immune cells^38^. β-adrenoceptor activation increases cAMP levels, and stimulation of β3-adrenoceptors may influence immune regulation and potentially enhance specific lymphocyte populations in obese mice^39,40^. The role of β3-adrenergic receptors (β3-ARs) in immune cells has been investigated, revealing that T-lymphocytes express both β2- and β3-Ars, with β3-AR expression predominantly increasing under stress^41^.

This study investigated mirabegron’s dual potential in managing OAB patients at risk of UTIs, evaluating its antibacterial activity in vitro and its immune-boosting effects in infected rats. Researchers assessed macrophage function and serum levels of IFN-γ, IgG, and IgM to explore its impact on innate and adaptive immunity. This marks the first time mirabegron has been tested against UTI-causing bacteria to improve infection outcomes and patient quality of life.

## Methods

### Chemicals

Mirabegron was manufactured by Astellas Pharma Inc. (Tokyo, Japan). The drug was dissolved in a physiological saline solution containing 5% cremophore (Sigma-Aldrich, USA) and 10% N, N-dimethylacetamide (Sigma-Aldrich, USA). MTT (3-(4,5-Dimethylthiazol-2-yl)-2,5-Diphenyltetrazolium Bromide) was purchased from ThermoFisherScientific, USA. INF-γ, IgG, and IgM were detected using specific ELISA kits (Glory Science Co., Ltd, China).

### Evaluation of the antibacterial activity of mirabegron

The antibacterial activity of mirabegron was evaluated using the culture media Mueller-Hinton agar for the agar diffusion method. The standard strains provided by ATCC^®^ (USA) of *Streptococcus agalactiae (S. agalactiae*,* NCTC 8181 [G19])*,* Staphylococcus aureus (S. aureus*,* NCTC 8532)*,* Pseudomonas aeruginosa (P. aeruginosa*,* PAO1)*, and *Escherichia coli (E. coli*,* NCTC 9001)* were taken from the culture plates of the respective microorganisms preserved on the nutrient agar and were kept at 4 °C in the Department of Microbiology, Faculty of Medicine.

Müller-Hinton agar^42^ is frequently utilized for testing antibiotic susceptibility as a microbiological growth medium. It generally has 2.0 g of beef extract, 17.5 g of casein hydrolysate, 1.5 g of starch, and 17.0 g of agar, all mixed in 1 L of distilled water. The pH was neutralized at 25 °C, and 5% sheep blood was used, sourced from a closely monitored herd of donor animals (Blood from healthy animals without antibiotics is certain). The antimicrobial activity was assessed using the agar well diffusion method^43,44^. The different bacteria were spread evenly across the Mueller-Hinton agar plates to completely cover them and achieve uniformity following incubation. 6 mm diameter wells were created on Mueller-Hinton plates using a sterile cork borer. Four consecutive dilutions of mirabegron resulted in concentrations of 10, 20, 30, and 40 µg/mL utilized for a pure substance. Each of the 4 wells on every bacterial plate received 100 µL of mirabegron. A fifth well contained 100 µL of ciprofloxacin at a concentration of 30 µg/ml to serve as a reference for its antibacterial effect on the bacterial plates. The plates were kept at 36 °C ± 1 °C, in aerobic conditions, for 24 h. Following 24 h of incubation, every plate was assessed for the presence of inhibition zones. The sterile broth without inoculation was made as a negative control, while acetic acid at 33% was used as a positive control. Using a caliper, the diameters of clear zones around the wells were measured. Experiments were conducted twice.

### Evaluation of the effects of mirabegron on macrophages and immunity

Regarding In vivo studies, male Wistar rats (200–250 g) were used in all experiments. Rats were purchased from the animal care house of the Faculty of Medicine. The rats were housed in stainless steel cages under a 12-hour light/dark cycle. Rats were given easy and free access to water and food. Twenty-four male Wistar rats were classified into 3 groups, 8 rats each. The first group of rats was employed as a control. The second group of animals was infected without treatment. Finally, the third group was infected and received oral mirabegron. All animals were utilized in accordance with ARRIVE guidelines (https://arriveguidelines.org). This work was approved by the Research Ethical Committee of the Faculty of Medicine, Assiut University (IRB. No. 04-2024-300424). All methods were performed in accordance with the relevant institutional guidelines and regulations.

The control and 2nd groups received the vehicle of mirabegron orally for 5 days. Regarding the 2nd and 3rd groups, animals were injected with a near-lethal dose of live *E. coli (NCTC 9001)* (1 mL of *E. coli* bacterial suspension, ca. 1 × 10^8^ CFU/mL) intraperitoneally once^45^. After 24 h, the 2nd group of rats received the vehicle of mirabegron orally for 5 days, while the 3rd group of rats received mirabegron by oral gavage (10 mg/kg/day)^46^ for 5 days. Twelve hours after the last administration of mirabegron on day 5, rats were anesthetized using intramuscular xylazine (10 mg/kg) and ketamine (90 mg/kg), and blood samples were taken from the carotid artery for analyzing INF-γ, IgG, and IgM. After animal were euthanized by cervical dislocation, the macrophages (5 × 10^5 cells/mL) were extracted from the peritoneal cavity by instilling and removing 8 ml of warm (37 °C) washing buffer (WB) (phosphate-buffered saline + 10% fetal bovine serum [FBS] + 100 ng/ml penicillin + 100 ng/ml streptomycin + 10 U/ml heparin + 20 U/ml DNase), and then cultured in 24-well plates with 200 µL of RPMI 1640 medium (10% FBS) for 2 h (37 °C, 5% CO2).

Evaluation of the cell viability was done using the Dimethylthiazoldiphenyl tetrazolium bromide (MTT) Assay. An MTT solution, made at a concentration of 5 mg/ml, was diluted tenfold using FBS-free culture medium. Subsequently, 100 µl of the diluted solution was added to every well. The plates were subsequently incubated at 37 °C with 98% humidity and 5% CO2. Throughout this time, viable cells transformed the soluble yellow MTT salt into insoluble purple formazan crystals via the action of mitochondrial succinate dehydrogenase. Following a 3-hour incubation, the culture medium was gently discarded, and 100 µl of dimethyl sulfoxide was introduced to each well to dissolve the formazan crystals. The measured color intensity, directly linked to cellular metabolic activity, was assessed at a wavelength of 570 nm on an ELISA reader. To verify the consistency of results, every assay was conducted three times. The percentages of cell viability were determined by taking the average absorbance of each group, dividing it by the average absorbance of the negative control group, and then multiplying the result by 100. An assay of Phagocytic Activity was performed. 100 µL of activated charcoal (1%) was added to 100 µL of cell suspension and incubated for 1 h at 37 °C. Following the incubation, the phagocytic activity was determined using the number of particles phagocytosed by macrophages and the average of one hundred cells^47^. One hundred randomly chosen macrophages were counted for phagocytic cells, and the average of the cells was expressed as a percentage of phagocytosis data. The phagocytic capability of macrophages was obtained as a percentage of phagocytosing macrophages and the number of phagocytosed particles per cell^48^.

The statistical analysis was carried out with GraphPad Prism, version 8.0.1 for Windows (San Diego, CA, USA, https://www.graphpad.com/updates/prism-801-release-notes). Normally distributed data were tested by one-way ANOVA with a post hoc Tukey’s correction. In all tests, a 95% confidence interval was used, for which *P* < 0.05 was considered a significant difference. Mean ± S.E. is how all data is portrayed. Microsoft Excel (2019) was used for analysis and graphical presentation of some results.

## Results

**Fig. 1:**
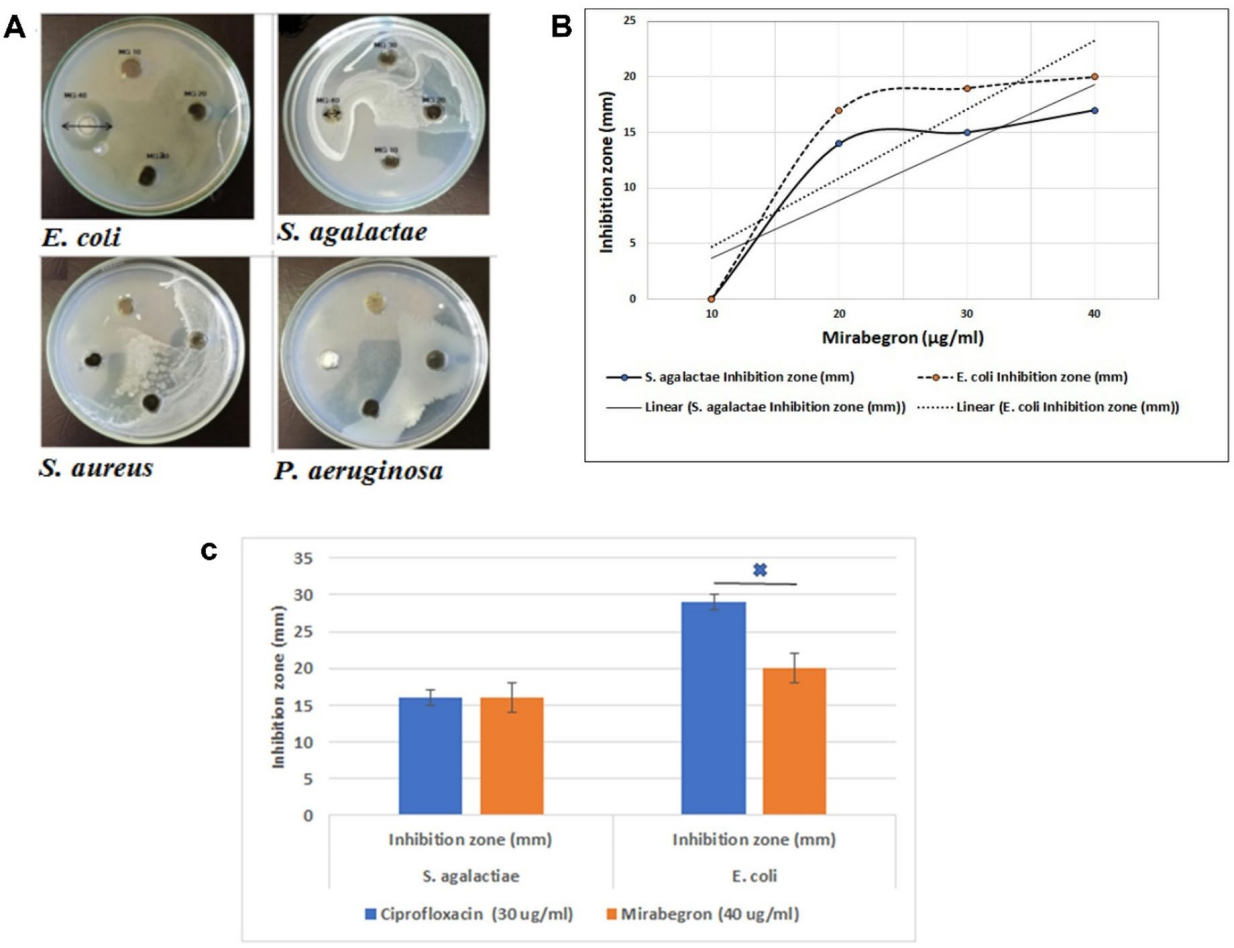
Well diffusion method shows (**A**) the zone of inhibition produced by mirabegron for *E. coli* and *S. agalactiae*, while no inhibition of growth of *S. aureus* and *P. aeruginosa* was observed; (**B**) Graphical presentation of the zone of inhibition in the well diffusion assay of mirabegron. MG: Mirabegron (on plate). (**C**) The inhibition zone of mirabegron and ciprofloxacin against the bacterial strains *E. coli* and *S. agalactiae.* Ciprofloxacin is more effective against *E. coli* than mirabegron.

The antibacterial effect of mirabegron was determined by measurement of the inhibition zone in millimeters. The bacterial inhibitory effect of mirabegron was observed for *S. agalactiae* and *E. coli*, and it was concentration-dependent (Fig. 1A and B). The highest inhibition zone was obtained at a concentration of 40 µg/mL. There was no inhibition zone detected for *S. aureus* or *P. aeruginosa*. In Fig. 1A, the zones of inhibition obtained with drugs were illustrated with plates. Figure 1B shows the graphic relation between the concentration of mirabegron and zones of inhibition for *E. coli* (20 mm) and *S. agalactiae* (16 mm). These values of inhibition zone were compared to those of ciprofloxacin, which were 29 mm for *E. coli* and 16 mm for *S. agalactiae*. This comparison was illustrated in Fig. 1C and shows a superior effect of ciprofloxacin over mirabegron for *E. coli*, but there is no difference for *S. agalactiae.*

**Fig. 2:**
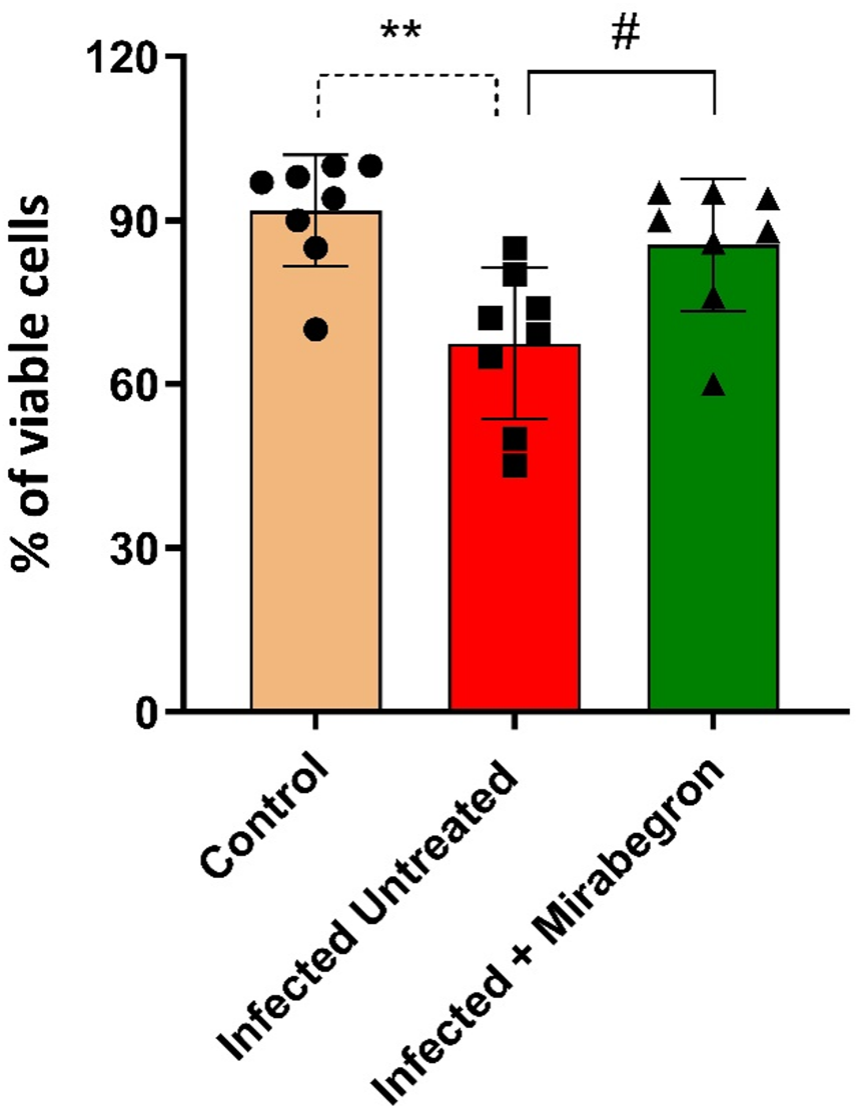
Cell viability of peritoneal macrophages by MTT method in *E. Coli*–induced sepsis and the effects of mirabegron treatment. The values represent the mean ± SEM of three independent experiments performed in duplicate. ** Significant difference compared to control rats (*p* < 0.01). # Significant difference compared to infected untreated rats (*p* < 0.05).

**Fig. 3:**
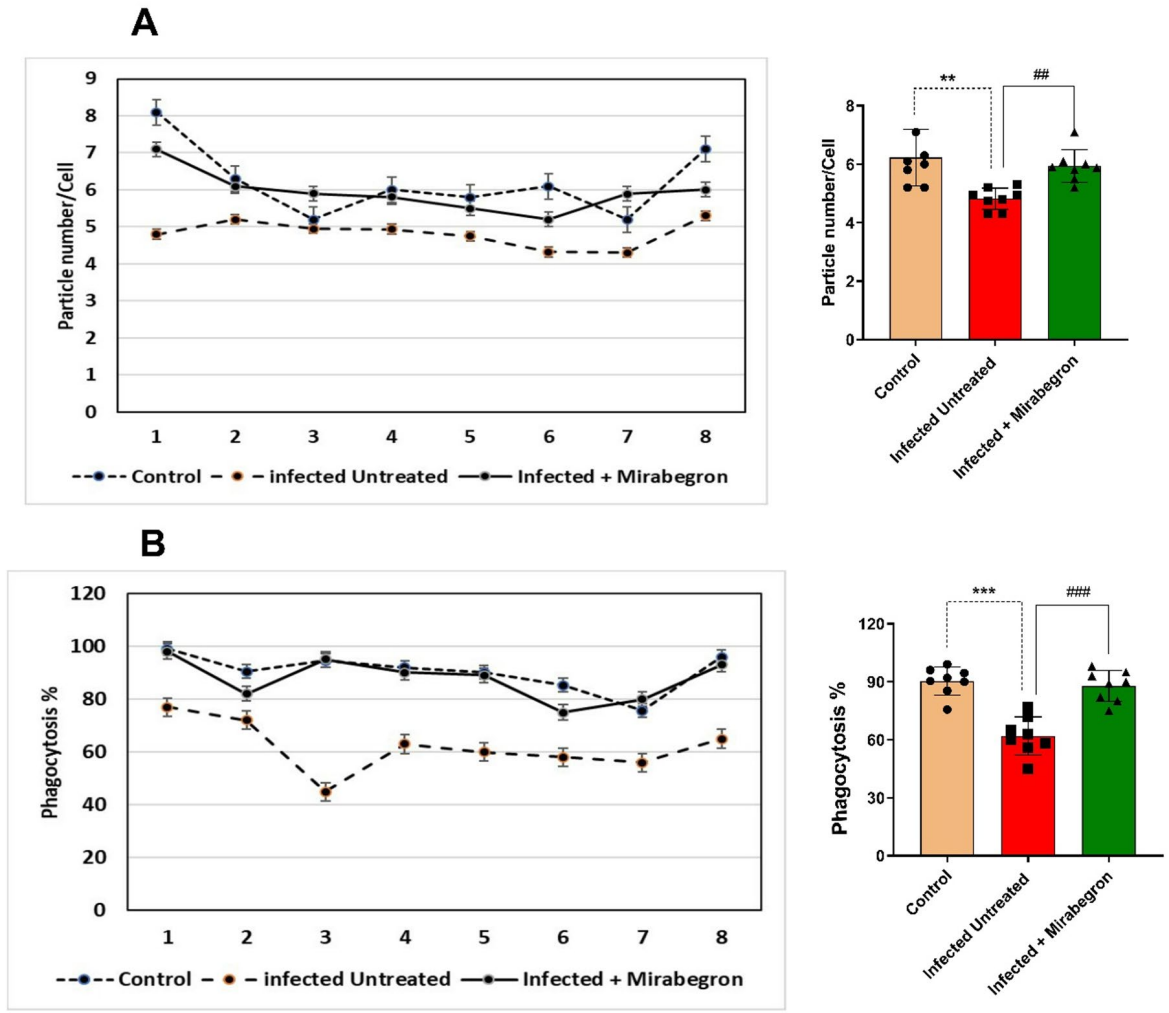
Phagocytic activity of peritoneal macrophages in *E. coli–induced* sepsis and the effects of mirabegron treatment. Phagocytic activity is represented by the number of phagocytosed particles (**A**) for each rat and the percentage of phagocytosis (**B**) of peritoneal macrophages in each group. Each value represents the mean ± SEM. ** Significant difference compared to control rats (*p* < 0.01). ## Significant difference compared to infected untreated rats (*p* < 0.01). *** Significant difference compared to control rats (*p* < 0.001). ### Significant difference compared to infected, untreated rats (*p* < 0.001).

Peritoneal macrophage viability was evaluated by MTT (dimethyl-thiazoldiphenyl-tetrazolium bromide) reduction; in control rats, viable cells were 91.75%. In Fig. 2, mirabegron administration led to a significant increase in macrophage activity as indicated by the reduction reaction of MTT (85.50%, P˂ 0.05). This significant effect became obvious when compared to rats with sepsis and untreated (67.50%). The phagocytic capability of the peritoneal macrophages was obtained as the percentage of phagocytosis and the number of phagocytosed particles to produce the phagocytic index. In control rats, the number of phagocytosed particles was higher than in infected, untreated rats (6.23 ± 0.34 particle number/Cell). When compared to untreated infected rats (4.82 ± 0.13 particle number/Cell), the administration of mirabegron increased both of percentage of phagocytosis (*p* < 0.001) and the number of phagocytosed particles (5.94 ± 0.19 particle number/Cell) (*p* < 0.01) (Fig. 3).

**Fig. 4:**
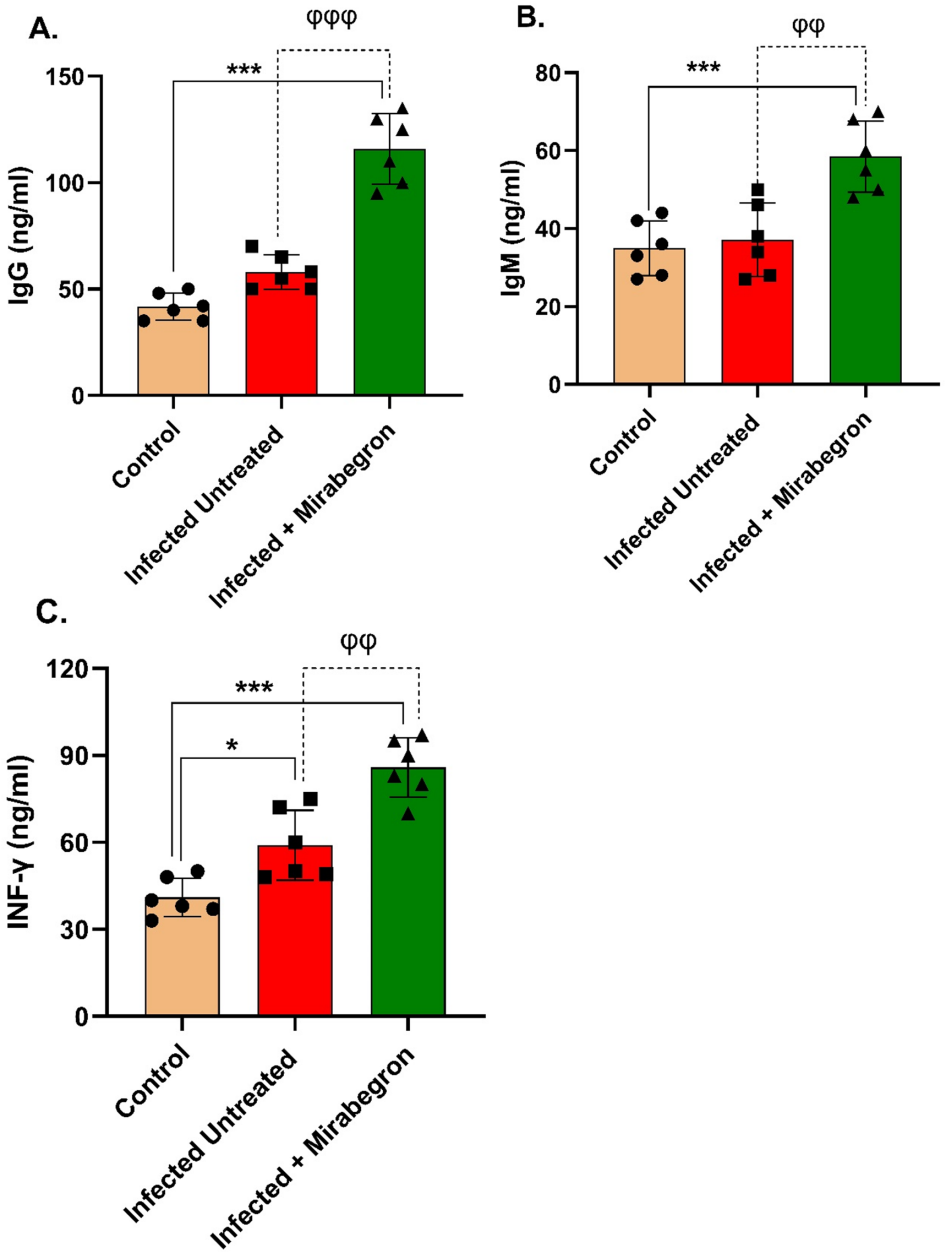
Effect of *E. coli*-induced sepsis and mirabegron on the serum levels of IgG (A), IgM (B), and INF-γ (C). Each value represents the mean ± SEM of 8 observations. *Significant differences at *P* < 0.05 vs. control group values. *** Significant differences at *P* < 0.001 vs. control group values. φφφ Significant differences at *P* < 0.001 vs. infected, untreated group values. φφ Significant differences at *P* < 0.01 vs. infected, untreated group values. IgG.

Oral administration of mirabegron increased the serum levels of IgG (133.20 ± 6.27 ng/ml), IgM (60.83 ± 4.32 ng/ml) and INF-γ (107.00 ± 5.27 ng/ml) when compared to control IgG (32.73 ± 6.85 ng/ml), IgM (26.47 ± 2.31 ng/ml) and INF-γ (37.87 ± 2.57 ng/ml) rats and infected rats untreated with mirabegron (UG) IgG (37.87 ± 2.57 ng/ml), IgM (31.37 ± 2.77 ng/ml) and INF-γ (75.43 ± 4.63 ng/ml) (Fig. 4A–C).

## Discussion

The overactive bladder (OAB) syndrome is defined by urgency, incontinence, frequency, and nocturia when no illness or other obvious cause is present. The primary method of treatment focuses on managing behaviour through reducing fluid intake, adhering to a urination schedule, bladder training, and strengthening the pelvic floor muscles. Anticholinergics and beta-3 agonists are oral drugs commonly prescribed to treat urgency and urgency incontinence. If other treatments do not work, the patient will be given injections of botulinum toxin A in the submucosa of the bladder^49,50^. Human detrusor relaxation in the bladder is primarily driven by β3-ARs, which account for approximately 95% of all β-AR mRNAs in this organ. Mirabegron reduces neurogenic detrusor activity and improves OAB by relaxing detrusors during the storage or filling phase of the micturition cycle^51^. Mirabegron has higher affinity and intrinsic activity for β3 adrenoceptors compared to β1 and β2 receptors. Mirabegron, the first β3-adrenoceptor agonist used to treat OAB, has been proven safe and efficacious in many randomized, placebo-controlled studies^52,53^.

Overactive bladder syndrome is a condition that occurs in the elderly. This condition is linked to worse health-related quality of life and a higher frequency of urinary tract infections^54^. Targeting inflammation and enhancing patients’ immunological responses is necessary to better treat and prevent urinary tract infections. For 12 weeks, males with lower urinary tract symptoms (LUTS) and OAB symptoms who received mirabegron in addition to tamsulosin outperformed a placebo and had higher tolerability^55^. Mirabegron was successful in treating male patients with chronic LUTS and OAB who did not respond to α1-adrenergic receptor blocker monotherapy. It had no negative effects on the voiding function. Furthermore, mirabegron supplemental treatment was deemed beneficial regardless of patient age^56^.

Mirabegron is an effective, dependable, and well-tolerated remedy for overactive bladder. When paired with anticholinergic medications, it provides more benefits without raising negative side effects^57^. Combining mirabegron with other drugs has proven to be a successful method of treating OAB symptoms without leading to major side effects. Despite the benefits of beta-3 adrenergic agonists in treating OAB, it is important for doctors to meticulously plan the pretreatment phase to enhance treatment results and compliance^58^.

This study aimed to assess the antibacterial properties and immunomodulatory impact of mirabegron. Initially, we assessed mirabegron’s potential to inhibit bacterial activity against various bacterial strains. We demonstrated that mirabegron exhibited noteworthy antibacterial effects on *E. coli* and *S. agalactiae* but did not show significant results against the other bacteria studied. Mirabegron is frequently prescribed for the common issue of frequent urination, a common symptom of urinary tract infections. *E. coli* is one of the primary bacteria that cause urinary tract infections. Mirabegron caused notable inhibition of *E. coli* growth in this research. This outcome shows potential for treating and managing urinary tract infection symptoms.

Some research indicated that urinary tract infection could be a potential side effect of mirabegron, whereas other studies did not find a link between mirabegron and UTIs^59^. The mechanism by which Mirabegron works in urinary tract infections is not completely known, and previous studies on the drug’s impact on isolated muscle preparations mainly relied on pharmacological data rather than functional urological data. The findings from clinical trials do not offer valuable information as they indicate no variation in UTI rates between patients who were given mirabegron and those treated with antimuscarinics in comparable patient cohorts^29^. Mirabegron inhibits bacterial growth, according to our in vitro findings. This debate might be attributable to the fact that the study was conducted in vitro, or it could be due to bacterial strain differences and medication concentration differences in vivo and in vitro, resulting in a distinct pharmacokinetic pattern. A pharmacovigilance study investigated the safety of mirabegron treatment with the help of the FDA Adverse Event Reporting System (FAERS) database. It helped improving mirabegron’s safety in clinical practice by providing useful data supporting its real-world safety^60^.

It has been suggested that peripheral β3-adrenergic receptors (ARs) generate pro-inflammatory cytokines and boost activation of immune cells^61^. Cyclic AMP, which acts as the messenger for beta receptors, is crucial in regulating the function of immune cells. The precise molecular process remains unclear. Certain research suggests that cAMP can have either inhibitory or stimulatory impacts on immune cell activation, cytokine release, and phagocytosis^62^. We suggested the possibility of mirabegron, which increases intracellular cAMP, which can modulate immune responses. Depletion of intracellular cAMP resulted in a significant inhibition of humoral and proliferative responses, indicating that cyclic AMP works as a positive regulatory signal for immune responses^63^. Administration of Mirabegron to mice with intracranial haemorrhage increased monocyte production and hematoma clearance, believed to be mediated by sympathetic activation^64^.

Mirabegron significantly improved peritoneal macrophage survival and engulfment ability during infection, according to our findings. Immunoglobulin IgG and IgM levels were also reported to be higher in the mirabegron-treated group after infection. Furthermore, the treated group saw a significant rise in IFN-γ levels. This suggests a rise in both innate and acquired immunity when using mirabegron. Likewise, Lamas et al.^32^ declared that beta3-adrenoceptor agonist drugs are not only used in the treatment of obesity but also play an important role in the regulation of immunity. He reported that beta 3-agonists improved lymphocyte proliferation, increased the number of lymphocyte subpopulations, and induced immunity as compared to the control group. The beta-adrenergic receptors found in immune cells have a crucial function in both the neuroendocrine and immune systems^65^. Treatment with a β3 adrenergic receptor antagonist led to a reduction in IFN-γ levels^66^. Furthermore, A study conducted in a laboratory setting found that Treg cell function was enhanced in T-cells taken from diet-induced obese mice after being treated with mirabegron^67^.

Claustre et al.^68^ discovered that beta 3 agonists play a significant role in modifying innate and adaptive immunological responses by stimulating peripheral beta 3 adrenergic receptors. Chemical sympathectomy reduced the increases in serum proteins and cytokine gene expression caused by sleep fragmentation^69^. Within the immune system, lymphocytes mostly express β-adrenergic receptors, whereas myeloid cells generally exhibit both α- and β-adrenergic receptors^70^. Since the majority of peripheral immune cells express β-adrenergic receptors^71,72^. It was proposed that blocking β-adrenergic receptors may lessen the inflammatory reactions caused by sleep fragmentation^73^. It was found that activating β3-AR reduces NADPH oxidase activation, leading to a decrease in ROS production by macrophages. In macrophages, the strong anti-inflammatory response was linked to the antioxidant effect. Moreover, β3-AR induces the expression of catalase in macrophages^74^. Mirabegron was observed to enhance adherence while maintaining a balance between efficacy and safety in individuals experiencing prostatitis symptoms^75^.

## Conclusion

Based on our results, mirabegron demonstrated significant antibacterial activity, particularly targeting *E. coli*. Additionally, it enhanced the survival and the ability of peritoneal macrophages to engulf particles. Furthermore, rats administered mirabegron exhibited increased serum concentrations of IgG, IgM, and INF-γ. As this medication has not been assessed previously, it is necessary to conduct further research in the future to either confirm or disprove our findings. It is extremely important to find a medication that not only manages the symptoms of OAB but also reduces bacterial load and enhances the immune response, since OAB patients are more prone to UTIs. The similarity between the symptomatic presentation of OAB and lower UTIs gave us the idea of trying mirabegron for UTIs, yielding its effects regarding bacterial growth and immune response as well as symptomatic relief.

## Limitations

It is crucial to study how mirabegron impacts bacterial growth to identify its potential mechanism and minimum inhibitory concentration. Furthermore, more research is needed to understand the impact of mirabegron on elements of the immune response. Therefore, mirabegron’s effects necessitate additional experimental designs and data collection. Moreover, additional research is necessary to clarify how mirabegron impacts urinary tract infection in a model involving bladder examination.

## Data Availability

The data determined and used during the present study are available from the corresponding author on reasonable request.

## Abbreviations

5-HT: HT 5-hydroxytryptamine
AM: Antimuscarinics
AUA/SUFU: The American Urology Association/Society of Urodynamics, Female Pelvic Medicine and Urological Reconstruction
BPH: Benign prostatic hyperplasia
IFN-γ: Interferon-γ
IgG: Immunoglobulin G
IgM: Immunoglobulin M
LUTS: Lower urinary tract symptoms
MIRA: Mirabegron
MTT: 3-(4,5-Dimethylthiazol-2-yl)-2,5-Diphenyltetrazolium bromide
OAB: Overactive bladder
PTNS: Peripheral tibial nerve stimulation
SNS: Sacral neurostimulation
UCPPS: Urinary chronic pelvic pain syndrome
UTI: Urinary tract infection
β-Ars: Beta adrenergic receptors
